# Progression of coronary calcification in healthy postmenopausal women

**DOI:** 10.1186/1471-2261-4-21

**Published:** 2004-12-01

**Authors:** Judith Hsia, Afifa Klouj, Anjana Prasad, Jeremy Burt, Lucile L Adams-Campbell, Barbara V Howard

**Affiliations:** 1 Department of Medicine, The George Washington University, Washington, DC 20037, USA; 2 Biostatistics Center Medical Center Unit, The George Washington University, Washington, DC 20037, USA; 3 Howard University Cancer Center, Washington, DC 20060, USA; 4 Medstar Research Institute, Hyattsville, MD 20783 USA

## Abstract

**Background:**

Coronary artery calcium score incrementally improves coronary risk prediction beyond that provided by conventional risk factors. Limited information is available regarding rates of progression of coronary calcification in women, particularly those with baseline scores above zero. Further, determinants of progression of coronary artery calcification in women are not well understood. This study prospectively evaluated rates and determinants of progression of coronary artery calcium score in a group of healthy postmenopausal women.

**Methods:**

We determined coronary calcium score by computed tomography and recorded demographic, lifestyle and health characteristics of 914 postmenopausal women, a subset of those enrolled in the Women's Health Initiative Observational Study. The 305 women with calcium score ≥10 Agatston units at baseline were invited for repeat scan. This analysis includes the 94 women who underwent second scans.

**Results:**

Mean age of study participants was 65 ± 9 years (mean ± SD), body mass index was 26.1 ± 6.1 kg/m^2^, and baseline calcium score was 162 ± 220 Agatston units. Mean interval between scans was 3.3 ± 0.7 years. A wide range of changes in coronary calcium score was observed, from -53 to +452 Agatston units/year. Women with lower scores at baseline had smaller annual increases in absolute calcium score. Coronary calcium scores increased 11, 31 and 79 Agatston units/year among women with baseline calcium score in the lowest, middle and highest tertiles. In multivariate analysis, age was not an independent predictor of absolute change in coronary calcium score. Hydroxymethylglutaryl coenzyme A reductase inhibitor (statin) use at baseline was a negative predictor (p = 0.015), whereas baseline calcium score was a strong, positive predictor (p < 0.0001) of progression of coronary calcification.

**Conclusion:**

Among postmenopausal women with coronary calcium score ≥ 10 Agatston units, rates of change of coronary calcium score varied widely. In multivariate analysis, statin use was a negative independent determinant, whereas baseline calcium score was a strong positive predictor of annual change in coronary calcium score.

## Background

Coronary calcium, assessed by computed tomography, strongly and independently predicts coronary risk [1-3]. Age is by far the most potent determinant of calcium score [4], although conventional risk factors also been associated with the extent of coronary calcification [4,5].

The rate of progression of coronary calcification appears to further stratify risk [6,7], but reports have been limited by sample size [8], retrospective design [6,8,9], inclusion of individuals with baseline calcium scores of zero [9,10] and limited interval between tomographic scans [8,10,11]. Further, not all studies adjusted for use of hydroxymethylglutaryl coenzyme A reductase inhibitors (statins), which have been reported to attenuate progression [9,12,13].

Calcium scores differ in men and women [4], but progression of coronary calcification has not been reported by gender, except for the Healthy Women Study, which only included women [10]. Of the 80 women in that cohort, 52 (65%) had calcium scores of zero at baseline. After mean follow up of 18 months, 47 of the 52 (90%) had no coronary calcium on repeat scan. Mean change for the 52 women was 0.4 Agatston units and median change was 0. Among the 28 women with measurable coronary calcium at baseline, mean change was 11 Agatston units for women with baseline calcium score 1–99, and 72 Agatston units for the 9 women with baseline calcium score ≥100.

In this study, we prospectively assessed the rate of progression of coronary calcification in an ethnically diverse group of healthy women with coronary calcium scores of at least 10 Agatston units at baseline, and identified independent predictors of progression.

## Methods

### Patient population

Study participants were a subset of women enrolled in the Women's Health Initiative Observational Study [14] at the George Washington University and Howard University/Medstar clinical sites between February 1995, and December, 1998. Women who joined this ancillary study provided informed consent in a form approved by the respective institutional review boards.

The entire Observational Study cohort comprises 93,676 women at 40 clinical sites. For this ancillary study, participants at the George Washington and Howard/Medstar clinics (n = 4435) were invited for computed tomography. Baseline scans were performed on the 914 women who responded to the invitation. Of these, 528 had no coronary calcium detected and 81 had calcium scores of 1 – 9 Agatston units. The remaining 305 women with calcium score ≥10 Agatston units were mailed a letter inviting them to have a second scan; African-American women received two mailings because of a historically lower response rate. This analysis includes the 94 women with serial scans, which were performed a mean of 3.3 ± 0.7 years after the baseline study.

### Variables

Participants provided data on a wide range of health variables including dietary habits, medical history and anthropometric measures. Questionnaire measures assessed self-reported hypertension, diabetes mellitus (excluding gestational diabetes), current smoking, high cholesterol requiring pills, postmenopausal hormone therapy, and family history of premature coronary disease (father with myocardial infarction at age 55 years or younger, or mother with myocardial infarction at age 65 or younger). Statin use at baseline was assessed by medication inventory.

Dietary fat consumption was assessed using a food frequency questionnaire based on instruments used in the Women's Health Trial [15]. Nutrient estimates from the food frequency questionnaire were similar to those from short-term dietary recall and from four-day food records [16].

Physical activity was assessed by questions on a frequency and duration scale of four walking speeds and three other types of activity classified by intensity (strenuous, moderate or light)[17]. For this analysis, we categorized women by the number of weekly episodes, at least 20 minutes in duration, of moderate or strenuous activity.

Plasma lipids were only measured in a 1% random subsample of Observational Study participants, so were not included as variables in these analyses.

### CT image acquisition and analysis

Images were acquired using an Imatron C – 150 scanner. Thirty contiguous 3-mm slides (100 ms/slice) were acquired during a single breathhold beginning 1 cm caudad to the carina. Each level was triggered by ECG in end-diastole (80% of R-R interval). Images were obtained with a 30-cm^2 ^field of view (pixel size, 0.586 mm). Images were analyzed by the Agatson method [18].

### Analysis

Descriptive statistics such as frequencies, percentages, means and standard deviations (SD) were used to describe the study population and to explore the relationships between coronary calcium score and several explanatory variables. Group comparisons were made by t test, chi square and, where appropriate, the Mantel-Hanzel chi square test. In Table 2, the p value is based on ranked scores because of variance heterogeneity. Annual change in coronary calcium score in Table 2 is adjusted for age using ANCOVA's least squares means.

**Table 2:** Annual change in calcium score

	Baseline calcium score		Annual change in calcium score			
			Unadjusted		Age-adjusted	% change
	(Agatston units)		(Agatston units/y)			
	mean (SD)	range	mean (SD)	range		
1 (n = 32)	32 (11)	13 to 51	11 (16)	-7 to 38	11	33%
2 (n = 30)	94 (39)	52 to 189	31 (31)	3 to 148	34	33%
3 (n = 32)	559 (292)	194 to 1236	79 (102)	-53 to 452	77	14%
p across tertiles			0.0001			

Determinants of annual change in calcium score were evaluated in a multiple linear regression model which included age, baseline calcium score, and statin use at baseline as independent variables (Table 4). In a separate model, hypertension was added. Age and statin use were selected as independent variables because they have consistently been identified as determinants of coronary calcification [12,13,19]. Baseline calcium score and hypertension were included because of their relationship to change in calcium score in Table 2 and 3, respectively. Analyses were carried out using *SAS System for Windows *v8.02.

**Table 3:** Risk factors by tertile of annual change in calcium score

	Tertile			p value
		
	1	2	3	
n	32	30	32	
	mean (SD)			
Age, y	64 (7)	65 (7)	67 (12)	0.55
Body mass index	25.1 (4.7)	26 (7.3)	27.1 (6.04)	0.45
% calories from fat	27 (6)	27 (7)	27 (10)	1.00
# days/week moderate-vigorous exercise	1.8 (2.2)	2.7 (2.6)	2.2 (2)	0.33
	%			
Hypertension	33	39	59	0.08
Diabetes	7	0	9	0.60*
High cholesterol	16	26	22	0.65
Current smoking	10	10	6	0.63*
Family history premature CHD	13	6	19	0.48*
Hormone use at baseline	48	42	44	0.87
Statin use at baseline	13	10	9	0.65*

* Mantel-Hanzel Chi Square used because of small cell sizes

**Table 4:** Multivariate analysis: Determinants of annual change in calcium score

	Parameter estimate	95% CI	p value
Age	0.4	-0.83, 1.63	0.52
Baseline calcium score	0.15	0.11, 0.20	<0.0001
Statin use at baseline	-43.95	-79.00, -8.88	0.015

## Results

Baseline demographic and health characteristics of the 94 women with serial scans are shown (Table 1). Among the 305 women with calcium score ≥10 Agatson units, characteristics of the 94 women with a second scan were similar to the 211 women without a second scan (data not shown), except that the former group was enriched for African-American women, 31/94 (31%) vs 11/211 (5%)(p < 0.0001 across ethnic groups).

**Table 1:** Comparison of women with serial scans vs women with calcium score <10

	Group A	Group B	
	mean (SD)		p value
N	94	609	
Age, y	65 (9)	61 (8)	<0.0001
Body mass index, kg/m2	26.1 (6.1)	25.9 (5.6)	NS
Baseline calcium score, Agatston units	162 (220)	0.5 (1.6)	<0.0001
% dietary calories from fat	27 (8)	26.5 (7.4)	NS
BP, mm Hg			
Systolic	126 (19)	119 (17)	0.001
Diastolic	75 (10)	73 (13)	NS
	No. (%)		
Ethnicity			<0.0001
White	58 (62%)	490 (80%)	
Black	31 (33%)	88 (14%)	
Asian/Pacific islander	3 (3%)	12 (2%)	
Hispanic	1 (1%)	11 (2%)	
Unknown	1 (1%)	8 (1%)	
Hypertension*	41 (44%)	146 (24%)	<0.0001
Diabetes mellitus	5 (5%)	7 (1%)	0.004
Current smoking	8 (9%)	24 (4%)	<0.05
Self-reported high cholesterol requiring pills	20 (21%)	76 (12%)	0.02
Hormone use at baseline	42 (45%)	366 (60%)	0.005
Statin use at baseline	9 (10%)	51 (8%)	<0.05
# days/week moderate/vigorous exercise	2.2 (2.3)	2.5 (2.4)	NS

Group A, women with baseline calcium score ≥10 Agatston units and serial scans;

Group B, women with baseline calcium score <10

* Self-reported hypertension or measured BP systolic >140 or diastolic >90

Table 1 compares the 94 women with serial scans, all with baseline calcium scores ≥10 Agatston units (Group A), with women whose baseline calcium scores were <10 Agatston units (Group B). Women in Group A were older, and more likely to report hypertension, diabetes, current smoking, and high cholesterol requiring pharmacologic therapy. Statin use was slightly more prevalent and postmenopausal estrogen use slightly less prevalent among women in Group A. Baseline calcium scores for white and African-American women were 278 ± 330 and 163 ± 214 Agatston units, respectively (p = 0.05).

Annual change in calcium score and age-adjusted change in calcium score are shown by tertile of calcium score at baseline (Table 2). Women with higher baseline calcium scores had greater absolute annual increase in both unadjusted (79 vs 11 Agatston units/year for women in the highest vs lowest tertile, p < 0.0001 across tertiles) and age-adjusted calcium score. Changes in calcium scores ranged from -53 to +452 Agatston units/year. Annual change in calcium score did not differ between white and African-American women (data not shown).

Coronary risk factors are shown by tertile of annual change in calcium score (Table 3). Body mass index, dietary fat consumption and physical activity were not different in women with more or less rapid progression of coronary calcification. Conventional risk factors, including age, cigarette smoking and diabetes, also demonstrated no significant trend across tertiles of progression in calcium score. Hypertension was reported somewhat more frequently by women with greater progression of coronary calcification (33% vs 59% in lowest vs highest tertile, p = 0.08).

In multiple linear regression analysis, age was not independently associated with annual change in calcium score (Table 4). Statin use was a weak negative predictor of progression (p = 0.015), whereas calcium score at baseline was a strong positive predictor of annual change in calcium score (p < 0.0001). Results were similar when hypertension, which had shown a non-significant trend across tertiles of change in coronary calcium score, was added to the model. Hypertension itself was not an independent determinant of progression (data not shown).

## Discussion

In this ethnically diverse group of Women's Health Initiative observational study participants, the rate of progression of coronary calcification was 33%/year among women with calcium scores between 10 and 190 Agatston units at baseline, and 14%/year for women with higher calcium scores. The rate of change in calcium score ranged widely in individual women, from -53 to +452 Agatston units/year. In multivariate analysis, statin use was negatively associated with progression of coronary calcification, whereas baseline calcium score was a strong and independent positive predictor of progression.

One strength of this analysis is the inclusion of a wide range of variables affecting atherosclerotic risk, including body mass index, physical activity, dietary fat consumption, postmenopausal hormone and statin use in addition to conventional risk factors. Another strength is the relatively long interval between scans, 3.3 years (mean), enhancing accuracy of the estimated rate of progression. This study includes only women, a group traditionally underrepresented in studies of coronary computed tomography [6,8,9,11], only those with measurable coronary calcium at baseline, and 37% non-white participants. Limitations include the sample size and absence of laboratory measures, such as lipids and glucose. These were performed only in a random 1% subsample of Observational Study participants, and consequently are not available for inclusion in this analysis.

The rate of progression of coronary calcification observed in this analysis is within the range reported by others [6,9,11]. Similarly, the lack of relationship between postmenopausal hormone use and progression of coronary calcification is consistent with prior reports [10,20], as is the observed inverse association of statin use with progression of coronary calcification [12,13].

Efforts to identify independent predictors of progression of coronary calcification have been limited, particularly in women. A retrospective study of 55 high-risk men identified baseline calcium score and Lp(a) as independent predictors of progression in a multivariate model which did not include statin use [21]. A prospective study of 87 men and 24 women identified baseline calcium score as a weak independent predictor (p < 0.05) of change in calcium score [11]. Smoking, hypertension, diabetes, age, plasma lipid levels, body mass index, prevalent coronary heart disease and use of lipid-lowering medications, aspirin, beta-blockers, angiotensin converting enzyme inhibitors, calcium antagonists or nitrates were not independent predictors of progression, although the ability to detect such relationships was limited in view of the sample size, which presents a similar limitation in this analysis.

In contrast, smoking was found to be an independent determinant of progression in a larger study of 311 men and 184 women, which also identified baseline calcium score as a potent predictor of progression [7].

Another limitation of this study was use of Agatston score, rather than calcium volume score. The latter provides better reproducibility [22], but was not in widespread use at the time baseline scans were acquired and was not available for the baseline scans in this study. The difference between Agatston and volumetric scores increases with the coronary artery calcium score. For example, among women aged 60–64 years, the 75^th ^percentile has been reported as 59 Agatston units or 42 volumetric units, and the 90^th ^percentile as 202 Agatston units or 163 volumetric units [23]. We cannot exclude the possibility that areas of coronary calcification may have consolidated through retraction during follow up, a phenomenon which may not have been accurately assessed using the Agatston score.

Calcium scores differ in men and women [24]; for example the 75^th ^percentile scores for 50–54 year old men and women are 99 and 3, respectively [4]. For 60–64 year old men and women, the 75^th ^percentile scores are 247 and 49, respectively. If baseline score is the major determinant of progression, a gender difference in rates of progression would be expected on this basis alone. In fact, male gender has been reported as an independent determinant of progression [7]. Whether rates of progression differ between men and women after adjustment for baseline score is uncertain.

Our observations raise several issues with regard to the potential use of coronary calcium score progression as either a clinical tool or an outcome for atherosclerotic intervention trials. First, the variance for this variable is high, both in our study and in others [7,9,12], limiting, at least to some extent, its value as a clinical predictor in individual patients and its appeal as an intermediate outcome. Second, stratification by statin use should be considered. Third progression should be adjusted for baseline calcium score.

## Conclusions

In an ethnically diverse cohort of postmenopausal women with coronary calcium score ≥10 Agatston units, rates of progression or coronary calcification vary widely. In multivariate analysis, statin use was inversely associated with progression, whereas baseline calcium score was a strong positive predictor of progression.

## Competing interests

The author(s) declare that they have no competing interests.

## Authors' contributions

JH study concept, design, data analysis, initial and final manuscript preparation. AK data analysis. AP and JB data acquisition and management, manuscript revision. LLA-C and BVH manuscript revision. All authors read and approved the final manuscript.

## Pre-publication history

The pre-publication history for this paper can be accessed here:


